# Listening to the Voices of Health Care Workers During the COVID-19 Pandemic: A Qualitative Study Providing In-Depth Insights Into Ethical and Individual Challenges

**DOI:** 10.1177/10497323241231521

**Published:** 2024-02-26

**Authors:** Barbara Buchberger, Heide Weishaar, Megan Evans, Rike Böttcher, René Umlauf, Swetlana Muminow, Eloisa Montt Maray, Nadine Muller, Souaad Chemali, Brogan Geurts, Hanna-Tina Fischer, Charbel El Bcheraoui

**Affiliations:** 1 Evidence-based Public Health, Centre for International Health Protection, Robert Koch 9222 Institute, Berlin, Germany

**Keywords:** ethical challenges, moral distress, dilemma, health care workers, COVID-19

## Abstract

In their daily practice, health care workers (HCWs) experience the effects of tensions between professional ethos and work realities, which can lead to ethical dilemmas. We aim to explore the ethical dilemmas that affected HCWs in Germany during the COVID-19 pandemic and to understand these in the context of the German health system. Between April and December 2022, we interviewed HCWs from various levels of care and key informants responsible for decisions related to HCWs in Germany. Three themes were identified in the data analyzed from 78 participants. The first highlighted the potency of pre-existing health system problems during the COVID-19 pandemic. The second captured the ethical dilemmas that were described as having arisen due to the tension between professional ethos and structural constraints. The third included factors related to increasing or diminishing the implications of ethical dilemmas. A lack of opportunities for HCWs to participate in political and managerial decisions was suggested to result in policies that do not meet the needs of HCWs and patients. Positive interpersonal interactions were described as helpful when coping with dilemmatic decision-making situations. In order to avoid negative consequences caused by unresolved ethical dilemmas, including moral distress, among HCWs, staff shortages and decision-making in the German health system urgently need to be addressed. HCWs’ working conditions regularly evoke ethical dilemmas, particularly during public health emergencies. Together with HCWs, decision-makers must develop new models for working in health care settings that are in line with HCWs’ professional ethos.

## Introduction

Medical, nursing, and therapeutic activities rely on people trusting others to take responsibility for their bodies and lives. In accordance with this responsibility, principles like “dignity of patients” (World Medical Association, 2017), “respect for human life,” and “duty” (South African Nursing Council, 2005) find an expression in the pledges of self-commitment of health care workers (HCWs). The work realities of HCWs, however, are not always in line with these principles. Professionals directly exposed to human suffering, such as doctors, nurses, and therapists, are likely to be affected by ethical dilemmas when they feel certain of the ethical course of action, yet are constrained from taking that action (Hamric & Blackhall, 2007). Resulting tensions can lead to dilemmas, which in turn can cause moral distress (Cribb, 2011; German Ethics Council, 2020; Kherbache et al., 2022). Ethical challenges and the difficulties of balancing professional codes of ethics with the institutional environment have, for example, been studied in the context of correctional mental health care (Segal et al., 2018). HCWs’ struggles in relation to handling the implications of ethical dilemmas have also been highlighted in several publications that have analyzed the situation of HCWs during the COVID-19 pandemic (Ehrlich et al., 2020; Hossain & Clatty, 2021). Particularly in the early stages of the pandemic, multiple factors including high workload and shortage of resources increased substantially and HCWs, who were placed at the forefront of the pandemic, were confronted with exceptionally high levels of mortality, human suffering, pain, and grief. This situation led to tensions between the realities of professional practice and HCWs’ professional ethos, tragic life and death decisions, and almost unsolvable dilemmas (German Ethics Council, 2020), as well as to high levels of moral distress (Čartolovni et al., 2021; Fernandez et al., 2020; Hossain & Clatty, 2021). In Germany, a large wave of intensive care nurses quitting their jobs during the pandemic meant that one in three beds in intensive care units had to be closed (Auffenberg et al., 2022). Based on this experience, experts have emphasized the need for professional, societal, and political debates and decisions about how to better support HCWs during pandemics (Walz et al., 2022). However, a comprehensive analysis of the specific ethical dilemmas faced by HCWs during the pandemic is currently lacking (Čartolovni et al., 2021; German Ethics Council, 2020). Also, little is known about which factors contributed to dilemmatic decision-making situations. This study seeks to fill these gaps by providing an in-depth analysis of the ethical dilemmas affecting HCWs during the COVID-19 pandemic in Germany, specifically focusing on capturing on HCWs’ personal accounts. Such an analysis should help decision-makers to identify possible solutions in order to prepare for future public health emergencies.

## Methods

We used focus group discussions with HCWs and individual interviews with HCWs and individuals involved in decision-making around health service provision, herein referred to as key informants (KIs). The analysis involved the integration of similar thematic coding, with findings being subsequently triangulated across different data sources for each theme.

We invited a wide range of HCWs and KIs to participate in the study. The HCWs came from different health care facilities (HCFs) in Germany (e.g., private practices, hospitals, and care homes), worked at various levels of health care, and were either invited to be interviewed individually or to participate in a focus group discussion (FGD). The HCWs included medical doctors, nurses, allied health workers, nursing students, and other staff working in HCFs during the pandemic. KIs that were interviewed included political decision-makers, health management personnel, academics, and other individuals with decision-making functions. All participants were required to be 18 years old or above and to provide voluntary informed consent.

Data collection took place from April to December 2022. Initially, HCWs were sampled through a parallel survey conducted in three randomly selected federal regions in Germany, selecting the volunteers who completed the survey and expressed their interest in participating in an interview or FGD. Due to a low survey uptake, the sampling approach was modified in May 2022 by including HCWs who worked in any federal region and including those who had not participated in the survey. To recruit additional HCWs, emails were circulated via professional networks and organizations. KIs were sampled by conducting an online search identifying potential interviewees from media reports commenting on the situation of HCWs during the pandemic. Purposive sampling was used to ensure a diverse selection of KIs from various regions, areas of work, genders, and roles. This included politicians, health facility managers, representatives of health professional associations, representatives of health care providers, academics, and others. KIs were initially contacted via email to provide them with comprehensive study information and assess their interest in participating. Information sheets and informed consent forms were shared with the participants before the interview, and follow-up communication was conducted twice. Interviews with KIs were conducted online via videoconference or face to face, while all HCWs’ interviews and FGDs were conducted online. HCWs received a 25-euro voucher as compensation, while KIs did not receive compensation. All interviews and FGDs were conducted in German and audio-recorded. Verbatim transcription was performed by an external company.

Topic guides relevant to the aim of the study were developed based on a literature review (Chemali et al., 2022) and covered personal experiences, challenges, coping strategies, factors supporting or hindering HCWs in coping with challenges, support needs, and recommendations for better support. Guiding questions were defined for each section and tailored to the data collection method (FGDs with HCWs, interviews with HCWs, and interviews with KIs). Topic guides were amended as data collection progressed to (1) account for emerging themes from previous interviews or FGDs and (2) clarify conflicting data.

All data were analyzed according to thematic content analysis (Braun & Clarke, 2022) using NVivo software 1.7. A codebook was developed deductively based on the topic guide and inductively according to additional topics that had come up during the interviews and FGDs and had been noted by the interviewee/facilitator or note taker either during or directly after the field work or by the researcher who had re-listened to the recording. A random sample of five interviews was independently coded by three researchers, coding was discussed, and the codebook was revised thoroughly according to consensus. One-third of the transcripts were double coded to ensure coding was consistent. Consistency checks were performed by a senior researcher. Ethical approval was obtained from the ethics committee of the nearby medical faculty, and data protection approval was obtained by the corresponding institutional department.

## Results

### Participants

Interviews were conducted with 18 representatives from management, policy, and academia and 17 HCWs. 43 HCWs took part in 10 FGDs, including therapists (*n* = 18), nurses (*n* = 14), doctors (*n* = 11), and other health care–affiliated professionals (*n* = 17) (Table 1).

**Table 1.** Socio-Demographics of Study Participants.

	Interviews		Focus group discussions (FGDs)
	Individuals involved in decision-making around health service provision (key informants, KIs)	Health care workers (HCWs)	
Number	*n* = 18	*n* = 17	*n* = 10
Duration (hour:minute)			
Total	16:30	15:47	11:33
Average (range)	00:55 (min 00:41–max 01:06)	00:55 (min 00:37–max 01:11)	01:09 (min 00:57–max 01:19)
Participants			
Number (range)	*n* = 18	*n* = 17	*n* = 43 (3–6)
Mean age, years (range)	Not available	48 (27–60)	47 (20–66)
Sex	Not available	Male = 5, female = 12	Male = 18, female = 25

During data analysis, three major themes were generated. Theme 1 related to pre-existing systemic problems and their perceived connection with ethical dilemmas during COVID-19. Theme 2 related to accounts of implications of ethical dilemmas during the COVID-19 pandemic. Theme 3 comprised both positive and negative factors influencing HCWs being exposed to ethical dilemmas.

### Pre-Existing Systemic Problems and Their Connection With Ethical Dilemmas During COVID-19

The data suggest a strong relationship between systemic problems in the German health system and resulting experiences of ethical dilemmas among HCWs. In particular, pre-existing staff shortages were mentioned by respondents, but also job dissatisfaction and mental resignation featured as problems that had existed prior to the pandemic. While systemic issues became evident during the pandemic, both HCWs and KIs reported that the underlying problems, and the related ethical challenges with implications for them, were not new but rather amplifications and exacerbations of longstanding issues within their professional lives. A focus group discussant stressed:

I think the pandemic has unmasked a lot […] what has been going wrong in the healthcare system over the last years, or decades, actually. (Doctor with specialization in anaesthesia, male, 57 years, tertiary care hospital)

Several HCWs described situations where they felt that moral pressure was exerted on them to go beyond their limits in order to make up for staff shortages. They recalled that supervisors appealed to their professional ethos, altruism, and collegiality to persuade them to work overtime. A study participant reported that when they declined working overtime and covering for colleagues, “the moral cudgels are swung, as they certainly always have been for ages: ‘You have to think about the patients, you have to think about your colleagues’” (study respondent). Similarly, several respondents reported that they had “a guilty conscience” (nurse, male, 49 years, tertiary care hospital) when they refused to work overtime. HCWs and individuals involved in decision-making around health service provision stressed that the shortage of staff and other pre-existing systemic challenges had become even more pronounced since the start of the pandemic, resulting in heightened pressure on HCWs. HCWs reported that cognitively, they understood that they needed breaks and rest time and were prone to make mistakes if they worked long hours, yet emotionally, it was difficult to stop working and take breaks as it felt like disappointing and abandoning both patients and colleagues and meant that managerial attempts to emotionally blackmail had to be resisted.

Several HCWs mentioned considerable frustration with the health system they worked in. They felt exploited and perceived the system to be functioning at the expense of the individual HCWs. Dissatisfaction related largely to structural issues like lack of appropriate financial incentives and responsible decision-making and to the fact that no effective measures were taken to improve the situation for those working on the ground. HCWs’ discontent with the lack of resolution of systemic problems was also acknowledged by KIs. An academic postulated that related frustrations would likely result in loss of staff:

I think [the fact that HCWs push themselves to the limit and thereby keep the deficient health system going] cause a lot of negative sentiment. And during the first wave I always said: “Care workers won’t just leave the sinking ship.” But if they get the feeling, during the second or third time, that someone keeps making leaks in the ship, then that might change. (University professor)

Health care workers confirmed that their frustration with the system led them to reconsider and, in some instances, quit their jobs or switch to a part-time work schedule. A senior doctor reported that with regard to quitting the job, the pandemic had “acted as a catalyst” (senior doctor in a university clinic). HCWs also reported that the structural deficiencies and the intensification of the workload during the pandemic had starkly highlighted the gap between professional ethos and daily work reality. Often, these experiences left HCWs “mentally or physically exhausted” and disillusioned (chairman of an institutional board). HCWs described that they felt helpless and unable to change the structural constraints that prevented them from following their professional ethos and doing their job in a way that they would have expected of themselves. Feelings of helplessness were also voiced by political decision-makers, health facility managers, and HCWs alike. HCWs further elaborated that a “feeling that you can’t do anything about it” frequently prevented them from even trying to initiate changing things and taking action (nurse, female, 50 years, secondary care hospital).

### Accounts of Ethical Dilemmas Affecting HCWs During the Pandemic

Respondents reported that it was challenging for HCWs to balance the need to protect themselves from risk of infection while continuing to treat patients. Both HCWs and KIs acknowledged that in fact, HCWs had been unable to fully protect themselves, particularly in the beginning before vaccines became available, and instead had often accepted the risk of infection and even the possibility of severe illness and death as part of their job.

Several HCWs expressed concerns about potentially infecting other patients or their loved ones. Remembering the early phases of the pandemic specifically, a HCW recalled, “this constant fear of having missed something, of accidentally dragging COVID into the facility” (health facility manager, female, 57 years, primary care facility). They also disclosed that “somehow you always perceived yourself as dangerous to others” (study participant).

Many HCWs recounted situations in which they had to make decisions and act professionally under very challenging conditions, encompassing working without having the necessary personal protective and medical equipment, lacking sufficient training and expertise to fulfil new tasks, and facing a lack of evidence to guide informed decision-making. Ethical challenges occurred particularly when patients needed end-of-life care. HCWs often reported that abiding by the COVID regulations meant that they had to compromise their professional ethos and obligation to provide a good standard of care. Challenges related, for example, to HCWs being unable to provide patient-oriented treatment due to regulatory constraints or being confronted with patients’ feelings of loneliness due to visiting restrictions, and these dilemmas were felt exceptionally strongly when dealing with critically ill and dying patients. Particularly in long-term care facilities, HCWs reported their attempts to act as substitutes for the lacking social contacts among residents yet were unable to do so due to time constraints and heavy workloads. Recalling the consequences of visiting restrictions in elderly care homes and their resulting attempts to also care for residents emotionally and socially, a HCW reported:

It was really, really bad for the patients, I must say. And also for us. We needed a lot of time with the patients. Because we also talked to the patient. […] And sometimes we brought something or the like. Or we took something away, a letter or something. So we tried […] to make it a bit more human. (Physiotherapist, female, 57 years, secondary care hospital)

### Factors Perceived as Influencing HCWs’ Experiences Due to Ethical Dilemmas

One aspect that was discussed as influencing one’s experience of being exposed to ethical dilemmas was professional ethos. Both KIs and HCWs stressed that HCWs have a motivation that “comes out of yourself” (study participant). They suggested that an intrinsic motivation to help patients was an inherent part of HCWs’ professional ethos and attitude toward work. Nursing and physiotherapy roles were described as a “relationship discipline” (physiotherapist, male, 60 years, primary care institution) and a “serving profession” (senior official, Ministry of Health of a federal state), respectively. These descriptions illustrate concepts of altruism, caretaking, and the commitment to prioritize patients’ interests. Accounts suggested that during the COVID-19 pandemic, HCWs encountered patients’ suffering with heightened intensity, which increased their desire to help and reinforced their dedication to their professional ethos. This served as a strong appeal to their professional ethos and fostered a clearer awareness of their obligations to follow ethical principles. This was also frequently perceived as a motivator in continuing their job despite adversities and risk of infection.

While both HCWs and KIs reported that a lack of overall understanding, unclear policies and guidelines, and contrasting regulations contributed to their experiences of being exposed to ethical dilemmas, the accounts of KIs often differed from those of the HCWs in relation to this topic. Those with decision-making powers primarily highlighted the lack of information and difficulties when having to make decisions under uncertain conditions and resource constraints. HCWs, on the other hand, felt subjected to arbitrary political and managerial decisions.

Health care workers working in nursing homes and in the primary care sector pointed out that the main focus of pandemic management was on hospitals and that “policymakers had completely neglected long-term care, […like…] facilities for the elderly, nursing homes, [or] facilities for the disabled” (KI_004, president of the German Association for Nursing Professionals). Regulations in nursing homes were reported to frequently produce ethical dilemmas and legal quandaries due to the fact that measures of isolation, quarantine, and facility closure (which, according to regulations, were implemented across all health care facilities) were perceived as the imprisonment of nursing home residents. Both managers and HCWs acknowledged that they practiced civil disobedience and disregarded official regulations to follow their professional ethos. Several HCWs reported that they had not worn personal protective equipment in order to provide good quality care or remain recognizable for patients with dementia. Similarly, managers reported that they had not followed legal orders to re-open health facilities in order to safeguard infection protection and avoid local outbreaks. Many HCWs concluded that decision-makers should have more trust in their individual responsibility, “common sense” (study participant), and ability to weigh up regulations, patient care, professional ethos, and work processes.

Concerning the structural misalignment between professional ethos and professional reality, HCWs expressed a lack of understanding toward the prioritization of their facility management, which they perceived as not patient centered:

They would rather pay for an ECMO (ventilator), or a bypass surgery, than […] for conversations that really serve to avert a catastrophe, or to prevent overtherapy, yes. And that’s what frustrates me […]. In some cases, there are clinics, I can tell you, where there is no ethical awareness at all. (Study participant)

Health care workers reported that those with decision-making powers were unaware of the actual work realities and needs of HCWs, with a study participant complaining that “the highest level, those who organize the work […] don’t know what we do and […] how we do it” (study participant). Hierarchical decision-making structures seemed to lead to a disconnect between managerial decisions and professional practice and a lack of opportunities for HCWs to promote or defend their values. HCWs often reported that they did not feel heard and seen, and that this was perceived to exacerbate the negative consequences of ethical dilemmas. In many statements, the HCWs interviewed made it clear that asking for their advice would have led to measures and regulations that would have been more in the patients’ best interests, as illustrated by the following quote:

I would wish that in our case the team […] are (sic!) heard, that […] the management or […] the head of nursing staff, that they simply also listen to us, accept suggestions for improvement from us. (Study participant)

Finally, positive interactions with colleagues and supervisors, valuable interactions with patients, and public recognition and support were reported to moderate the effects of ethical dilemmas and alleviate related distress. On the other hand, conflicts within the team, a lack of managerial support and public recognition, and conflicts with patients and relatives were reported to have detrimental effects. The general lack of collegiate interaction and exchange due to contact restrictions, shortages of staff, and prioritization of direct patient care was perceived as detrimental. Lack of interaction and failed conflict resolution was often reported to be a result of the high workload. At large, however, respondents reported having positive interactions with patients and within their teams, and also of appreciative collaboration between the different actors involved in the COVID-19 response. It was frequently reported that feelings of being “in the same boat” (senior doctor, university hospital), having “a common goal” (study participant), and following the same ethos fueled a sense of community, and that good collaboration had helped them to master major challenges and endure dilemmas. Various HCWs reported on initiatives and creative solutions that had come from within their teams to strengthen the sense of community which had also helped to deal with suffering and grief and alleviated the negative implications of ethical dilemmas. A senior doctor reported that in the early phases of the pandemic, they had established a routine of having a debriefing session after distressing events:

I had experienced this once in an intensive care unit after a very stressful situation years ago, when I was still quite fresh in my job and found it so positive. […] That’s why I started out, for example, with the habit that after a resuscitation, after every resuscitation or after a death that was fulminant in a young patient, we all sit down together and have coffee. And everybody speaks again and expresses their feelings if he or she wants to. (Senior doctor, university hospital)

The data suggest that engaging in discussions about their experiences and challenges with colleagues allowed HCWs to step back from work routines, which, in addition to others’ understanding and respect for their specific needs, helped them to cope with a demanding work environment and dilemmatic decision-making situations.

## Discussion

This paper provides in-depth insights into the experiences of those working in health services related to professional ethos and ethical challenges during the COVID-19 pandemic. Our analysis highlights numerous challenges faced by HCWs during the pandemic and sheds light on the vulnerabilities of the health care system in Germany. The study illustrates that the pre-existing systemic weaknesses, the specific and frequently changing pandemic regulations, and the resulting increase and compression of workload put a massive strain on HCWs and made it hard for them to follow professional ethos. The analysis suggests that the disparity between professional ethos and realities of professional practice further exacerbated during the pandemic, leading to ethically dilemmatic situations. Previous literature shows that failure to develop solutions to overcome dilemmas leads to moral distress which can manifest itself in symptoms such as frustration, anger, or feelings of guilt (Epstein & Hamric, 2009), which were also expressed by the HCWs in our study. Existing studies on moral distress are predominantly focused on nurses, suggesting that they experience higher levels of moral distress than doctors (Fourie, 2017; Hamric, 2012; Hamric & Blackhall, 2007; McCarthy & Deady, 2008). As investigated by several researchers, this is due to specific constraints caused by their position in medical hierarchies (Kherbache et al., 2022). At worst, the consequence is quitting, which further aggravates personnel shortages and increase in workloads. However, participants also reported that a number of work-related factors, notably positive professional interactions and participatory approaches to decision-making, helped to cope with dilemmatic situations, thus providing indication of which factors would be worth tackling to reduce the risk of moral distress.

The study puts the focus on existing health system challenges related to demographic change, skills shortage, and suboptimal working conditions. Our analysis suggests that human resources were particularly stretched in nursing during the pandemic, as the German nursing sector had been at its limits due to insufficient personnel for some time prior to the pandemic (Auffenberg et al., 2022). Persistent staff shortages are related to permanent compression of workload, which leads to negative effects on HCWs’ physical and mental health, participation in social life, substandard quality of care, and job dissatisfaction (Auffenberg et al., 2022; Greenberg & Tracy, 2020). Considering the reports of nurses presented in this study, which illustrate the immense burden they experienced during and prior to the pandemic, it is unsurprising that 35% of all nursing staff contemplate leaving the profession multiple times a year and 40% contemplate switching employers (Auffenberg et al., 2022). According to a study by the German Professional Association for Nursing Professions, nurses are likely to consider re-entering the profession if more favorable working conditions can be assured, for example, binding duty rosters, no regularly scheduled overtime, not having to cover additional shifts when off duty, no split shifts, and being able to finish work on time with a clear conscience (Auffenberg et al., 2022). In our study, HCWs also reflected on the conflict between the working conditions which make it hardly possible to meet their own demands for good patient care and intrinsic motivation and professional ethos. They also reported about colleagues who had resigned or reduced their working hours due to work circumstances in order to resolve these conflicts. Such resignations further aggravated the lack of skilled workers and increased workload, inevitably resulting in a deterioration in the quality of care and thus fueling a vicious cycle.

The analysis shows that HCWs had to endure a conflict of duties, for example, in relation to having to treat patients yet needing to protect themselves from infection despite insufficient protective equipment. Dealing with the fact that one could unintentionally infect patients or loved ones was comparably dilemmatic. Examples also related to conflicts of conscience caused by the need to take breaks while being faced with a large number of patients and high workload and being aware that good quality of care could only be provided when rested. Such instances where HCWs had to act despite conflicting duties frequently led to unease.

The tension caused by conflicting duties that HCWs experienced was illustrated through the idioms and metaphors they used. The ship metaphor of one boat in which everyone sits together was frequently mentioned to depict the pandemic situation. This metaphor stresses three distinct aspects: first, it signifies the exposure to unpredictable and dangerous (weather) conditions, symbolizing the challenges posed by the pandemic; second, it emphasizes a strong sense of community as everyone is united in one place; and third, it signifies a shared objective (of those sitting in the boat), namely, providing care and support for those who are suffering. The use of such metaphors allows for different interpretations: one is that the language of medical science is to be more or less consciously abandoned, as in the conversation with patients, so that a flight into figurative language takes place because the mental shock about the overall situation cannot be expressed otherwise. Another explanation might be that as an internal constraint, the words or the ability for ethical reflection is missing (Kherbache et al., 2022).

A main finding of our analysis is the importance of a sense of community, which seems to be able to alleviate the negative consequences of ethical dilemmas. These findings are in line with the literature which shows that HCWs’ ethical identities are partly constituted by their membership in a professional community “because this is a community through which he or she negotiates and achieves his or her self-identity” (Cribb, 2011). The HCWs who were interviewed as part of this study reported that pursuing a common goal, professional exchange, peer support, and positive interactions strengthened them in handling dilemmatic decision-making. Beyond this, our study also points to a number of risks that are inherent to HCWs’ desire to work collaboratively for a common cause. Our analysis suggests that HCWs’ prioritization of patients’ needs over their own sometimes led to a lack of self-care and even self-exploitation. HCWs also reported that their dedication to supporting patients was exploited by health facility managers and political decision-makers. The latter were perceived as making decisions without considering potential negative impact on HCWs. HCWs’ reports of supervisors appealing to professional ethos and altruism or paying HCWs a bonus in order to get them to work overtime have to be assessed very critically as an attempt to exercise hyper morale. Appealing to HCWs’ professional ethos while not showing consideration for HCWs’ needs and rights can be seen as unethical. Being able to demand professional ethos is just as much a misunderstanding as wanting to generate intrinsic motivation through extrinsic motivation, which is rather compromising professional ethos. Professional ethos cannot solely apply to those working at the patient’s bedside but has to extend to those who make decisions that affect HCWs and their work realities, including health facility managers and political decision-makers.

Our study draws attention to a clash of managerial values and the values that make up the professional ethos of HCWs. Respective conflicts between profit-oriented managerial decisions and patient-centered care are a structural weakness of the health care system and cannot be easily reconciled. Such differences in values are exacerbated by lack of participation and hierarchical decision-making. If the corporate identity of an institution is dominated by the managerial pursuit of profit, and those working directly with patients cannot make themselves heard, frustration and other negative implications, including moral distress, will grow. In line with this, the study participants reported that their perceived powerlessness in the face of the overall situation resulted in distress. They emphasized that HCWs felt powerless to initiate change in their work environment due to a perceived lack of influence in managerial or political decision-making. These findings are in line with previous literature which states that the perception of having no voice or being overpowered by management can be a major contributor to moral distress (Austin, 2012). The perceived violation of basic values or duties means that individual moral concerns may go unaddressed, leading to an erosion of moral integrity which is part of professional ethos (Epstein & Hamric, 2009). Epstein and Hamric (2009) describe moral distress as a powerful negative phenomenon due to the violation of one’s core values and obligations. They also say that those who are “morally distressed feel as though they must behave in an ethically inappropriate manner, in part because their views have not been heard” (Epstein & Hamric, 2009). The findings also resonate with previous research that highlights the importance of understanding and accounting for the unique perspectives of health care workers when improving and ensuring patient-centered care (Balbale et al., 2015).

While highlighting clashes between working conditions and professional ethos, our study also points to some possible solutions for addressing these challenges. Opportunities for participation in decision-making, advocating for patients’ interests, and increased two-way communication between management and staff as well as within interdisciplinary teams were mentioned as positive examples that helped HCWs to have a voice and improve their work situations. In fact, opportunities for participation in managerial and political decisions could be understood as a continuation of a care process which is based on shared decision-making and patient-centered care. Such approaches to care provide room for interaction and facilitate that HCWs can act in the best interest of the patients and their relatives (Kherbache et al., 2022). Subsequently, giving HCWs the possibility to advocate for the patients’ interests according to professional ethos while also accounting for their own needs cannot be overstressed. One way to give HCWs a stronger voice might be to abandon the belief that small-scale regulations improve people’s work; instead, deregulation, for example, by reducing documentation obligations and corresponding audits and sanctions, should be introduced and trust increased in HCWs and their ability to act according to their professional ethos, a wish that was frequently expressed in our study. If HCWs are not given the opportunity to participate in decision-making, feelings of powerlessness and of providing care that is not in the best interest of the patient and in accordance with professional ethos are likely to increase moral distress and, eventually, moral residue. In addition to better communication and participatory interaction between HCWs and decision-makers, good communication within teams of HCWs within one unit, across different departments of a health care facility and across different health facilities, can help to cope with dilemmatic situations. The fact that several HCWs recalled instances during the pandemic where their voices had been heard, for example, when they had been asked for their opinion about the practicability of facility-specific policies, can be interpreted as hopeful signs of successful communication and can point to ways in which communication and decision-making could be improved in the future. The instances of unresolved ethical dilemmas that were reported in our study, however, indicate that there is still a general lack of discussions on ethics that include the perspectives of HCWs (Epstein & Hamric, 2009; Oh & Gastmans, 2015). In order to reduce and prevent moral distress in the context of challenging work realities, formats for exchange on ethical issues and peer counselling and participatory decision-making should be promoted.

While our study provides in-depth insights into HCWs’ perceptions of ethical challenges, a number of limitations have to be noted. First, the cross-sectional study design did not allow insights on changes over time and the current situation. This might be crucial as moral distress, if not resolved in a satisfactory way, can result in moral residue (Epstein & Hamric, 2009). Subsequently, a “crescendo effect” may occur due to repeated and unresolved instances of moral distress by which the affected person’s moral residue expands (Epstein & Hamric, 2009; Kherbache et al., 2022). Second, HCWs’ accounts of distress were not validated via an objective measurement of moral distress (Dudzinski, 2016; Tian et al., 2021). Rather, accounts of being exposed to ethical dilemmas and resulting distress were raised by interviewees without any prompts, which means that the analysis presented in this paper is based on an inductive analysis of participants’ responses. Third, selection bias was introduced by identifying KIs from media reports commenting on the situation of HCWs during the pandemic. In addition, self-selection of HCWs limits the breadth of perspectives presented.

## Conclusions

Our study shows that the working conditions of HCWs in Germany regularly evoke ethical dilemmas and that these were aggravated during the COVID-19 pandemic. Unresolved ethical dilemmas can result in moral distress and eventually illness and job resignation. HCWs’ reports about compromising care due to structural constraints indicate the negative consequences of such unresolved dilemmas at the health system level and the inherent risk for patients’ health and wellbeing. Therefore, decision-makers need to urgently develop new models for working in health care settings, specifically during public health emergencies. These models should be developed through participatory approaches and include HCWs at every stage of the process, in order to account for the needs of patients and those who are in close contact with them and are in line with HCWs’ professional ethos.
